# Adherence to a Mediterranean Dietary Pattern Is Associated with Higher Quality of Life in a Cohort of Italian Adults

**DOI:** 10.3390/nu11050981

**Published:** 2019-04-29

**Authors:** Justyna Godos, Sabrina Castellano, Marina Marranzano

**Affiliations:** 1 Department of Medical and Surgical Sciences and Advanced Technologies “G.F. Ingrassia”, University of Catania, 95123 Catania, Italy; justyna.godos@student.uj.edu.pl; 2 Department of Educational Sciences, University of Catania, 95124 Catania, Italy; sabrinacastellano@hotmail.it

**Keywords:** Mediterranean diet, quality of life, mental health, fruit, vegetable, dairy, nuts, fish, whole-grain, food groups

## Abstract

Background: The observed rise in non-communicable diseases may be attributed to the ongoing changes of urban environment and society, as well as greater awareness of health-related issues and subsequent higher rates of diagnosis, which all contribute to the overall quality of life. The aim of the study was to test the association between adherence to the Mediterranean dietary pattern and self-reported quality of life in a cohort of Italian adults. Methods: The demographic and dietary characteristics of 2044 adults living in southern Italy were analyzed. Food frequency questionnaires (FFQs) and a Mediterranean diet adherence score were used to assess dietary intake. The Manchester Short Appraisal (MANSA) was used to assess self-rated quality of life. Multivariate logistic regression analyses were used to test the associations. Results: A significant linear trend of association was found for the overall quality of life and adherence to Mediterranean diet score. All of the components of the MANSA, with the exception of self-rated mental health, were individually associated with higher adherence to this dietary pattern. Conclusions: Adherence to a healthy dietary pattern is associated with the measures of better overall perceived quality of life.

## 1. Introduction

Modern society and “Westernized” lifestyle has been negatively associated with the mental and physical health of the population. The observed rise in non-communicable diseases may depend on ongoing changes of urban environment and society, as well as a greater awareness of health-related issues and subsequent higher rates of diagnosis [1]. Quality of life has been generally used as an outcome of interest to evaluate the general condition of health and well-being of a person [2]. Self-perceived health status may obviously depend on current health conditions; more importantly, it has been found to be a close predictor of mortality [3,4,5]. In this context, factors that influence health-related quality of life of general population have paramount importance for their potential preventive role in improving health. Among major risk factors, poor nutrition has been considered as the main contributor to non-communicable diseases, including mental disorders [6]. There are indirect links between dietary patterns and mental disorders, including socioeconomic circumstances, obesity, and occurrence of conditions that might be associated with, or drivers of, chronic conditions (i.e., cardiovascular disease) [7]. However, investigating the relation between the risk factors that affect self-perceived health might be helpful to provide proof for potential underlying mechanisms.

The Mediterranean diet appears to be protective against cardio-metabolic diseases [8,9,10], neurodegenerative disorders [11,12], and certain cancers [13] amongst the most studied dietary patterns. The Mediterranean dietary pattern does not stand for a unique pattern of foods; rather, refer to the consumption of foods that characterize the dietary habits of the individuals living in the Mediterranean coasts [14,15]. The higher consumption of majority of plant-derived foods, such as fruit, vegetable, legume, and whole-grains, using olive oil as main source of fat, moderate alcohol consumption, moderate intake of fish and dairy products, and limited intake of meat and highly processed foods [16] represent the key features of this dietary pattern. Moreover, the beneficial effects of Mediterranean diet may relay on the high content in antioxidants and anti-inflammatory compounds, which have been hypothesized to play a role in the prevention of both physical and mental disorders [17]. Such findings suggest that adherence to the Mediterranean diet may be associated with better health and, consequently, better perceived health. In fact, previous observational studies showed that a higher adherence to the Mediterranean diet was associated with a better health-related quality of life [18,19,20,21,22]. Aspects that are related not only to physical health, but also to mental and psychosocial health, have underlined the importance of engagement in social activities as the potential determinant of better collective well-being [23,24]. Individuals living in the Mediterranean area may benefit of a characteristic way of living, in particular, through better social interactions with their next of kin and their community, including the frequency of contacts [25], network size [26], and relationships that are derived from personal choice [27], which is associated with positive outcomes and higher quality of life [28]. However, the number of studies investigating the association between Mediterranean diet and quality of life is limited and the topic may have further application for public health purposes, is therefore worth further examination. Thus, this study aimed to test the association between self-reported quality of life and the adherence to the Mediterranean dietary pattern in a cohort of Italian adults.

## 2. Materials and Methods 

### 2.1. Study Population

The study sample was constituted of participants of the Mediterranean healthy Eating, Aging, and Lifestyles (MEAL) study, an observational investigation that is primarily focused on nutritional habits and their relation with a cluster of lifestyle behaviors that characterize the classical Mediterranean lifestyle. The study protocol with the rationale, design, and methods have been described in detail elsewhere [29]. Briefly, the cohort consisted of 2044 men and women older than 18 years old (mean and standard deviation 48.1 ± 17.5 years) that were recruited in the urban area of Catania, which is one of the largest cities in the east coast of Sicily, southern Italy during 2014–2015. A random sample of individuals registered in the records of local general practitioners was invited to participate. The sampling technique included stratification by municipality area, age, and sex of inhabitants, and randomization into subgroups, with randomly selected general practitioners being the sampling units, and the individuals registered to them comprising the final sample units. Out of the 2405 individuals invited, the final sample size was 2044 participants (response rate of 85%). All of the study procedures were carried out in accordance with the Declaration of Helsinki (1989) of the World Medical Association. Participants provided written informed consent and the ethics committee of the referent health authority approved the study protocol.

### 2.2. Data Collection

Data regarding demographic (i.e., age, sex, educational and occupational level) and lifestyle characteristics (i.e., physical activity, smoking and drinking habits) were collected. The educational level was categorized as (i) low (primary/secondary), (ii) medium (high school), and (iii) high (university), while occupational level was categorized as (i) unemployed, (ii) low (unskilled workers), (iii) medium (partially skilled workers), and (iv) high (skilled workers). Physical activity level was evaluated through the International Physical Activity Questionnaires (IPAQ) [30], which comprised a set of questionnaires (five domains) on time that is spent being physically active in the last seven days that allow for categorizing physical activity as (i) low, (ii) moderate, and (iii) high. Smoking status was categorized as (i) non-smoker, (ii) former smoker, and (iii) current smoker. Alcohol consumption was categorized as (i) none, (ii) moderate drinker (0.1–12 g/day), and (iii) regular drinker (>12 g/day). Anthropometric measurements were collected using standardized methods [31]. Standing height was measured through a scale stadiometer to the nearest 0.5 cm and weight to the nearest 0.1 kg without shoes, with the back square against the wall tape, eyes looking straight ahead, and with a right-angle triangle resting on the scalp and against the wall. Body mass index (BMI) was calculated, and the patients were categorized as under/normal weight (BMI < 25 kg/m^2^), overweight (BMI 25 to 29.9 kg/m^2^), and obese (BMI > 30 kg/m^2^) [32]. Information from measurements was integrated with general practitioners computerized records in order to ascertain the cases of hypertension, diabetes, dyslipidemia, previous cardiovascular disease event, and cancer (all diagnoses were confirmed by a specialist before being registered).

### 2.3. Dietary Assessment

Long and short food frequency questionnaires (FFQs) specifically developed and validated for the Sicilian population were used to collect dietary data [33,34]. The FFQs consisted of 110 food and drink items that were representative of the diet during the last six months. The participants were asked how often, on average, they had consumed foods and drinks included in the FFQ, with nine responses ranging from “never” to “4–5 times per day”. The intake of food items characterized by seasonality referred to consumption during the period in which the food was available and then adjusted by its proportional intake in one year. A total of 1937 individuals were included in the analyses for the present study after the exclusion of 107 entries with unreliable intakes (<1000 or >6000 kcal/day, controlled case by case, and being validated due to missing food items or unreliable answers).

### 2.4. Adherence to the Mediterranean Diet

A score derived from the literature assessed the Mediterranean diet adherence: briefly, a scoring system (the MEDI-LITE score) was built while taking into consideration median (or mean) values that were reported in selected studies and two standard deviations to determine three different categories of consumption for each food group [35,36]. For food groups that typically characterize the Mediterranean diet (fruit, vegetables, cereals, legumes, and fish), two points were given to the highest category of consumption, one point for the middle category, and 0 point for the lowest category. Conversely, for food groups that are not typical of the Mediterranean diet (meat and meat products, dairy products), two points were given for the lowest category, one point for the middle category, and 0 point for the highest category of consumption. For alcohol, categories that are related to the alcohol unit (1 alcohol unit = 12 g of alcohol) were used by giving two points to the middle category (1–2 alcohol units/day), one point to the lowest category (>1 alcohol unit/day), and 0 point to the highest category of consumption (>2 alcohol units/day). The final score comprised nine food categories (including olive oil), with a score ranging from 0 point (lowest adherence) to 18 points (highest adherence).

### 2.5. Quality of Life Assessment

The Manchester Short Assessment of Quality of Life (MANSA) [37], an instrument that consists of 12 subjective items with a seven-point Likert scale (from “could not be worse” to “could not be better”) and four yes/no questions related to objective aspects of social life, assessed quality of life. The instrument assesses satisfaction with life as a whole and across several specific domains (including employment, financial situation, friendships, leisure activities, accommodation, personal safety, people living in household/living alone, sex life, relationship with family, and physical and mental health). An overall satisfaction with overall quality of life was arbitrarily defined as being in the highest quartile of the MANSA score (>70 points).

### 2.6. Statistical Analysis

The variables were examined for normality and skewness using the Kolmogorov–Smirnov test. The categorical variables are presented as frequencies of occurrence and percentages; differences between groups were tested using the Chi-squared test. The differences in background characteristics across participants grouped into quartiles of the MANSA score were tested. The relation between adherence to the Mediterranean diet and quality of life was tested through multivariate logistic regression analysis (when considering quartiles of the Mediterranean diet adherence score) adjusted for baseline characteristics (age, sex, marital, educational and occupational status, smoking and alcohol drinking habits, physical activity level, health status, and energy intake). All of the reported *P* values were based on two-sided tests and compared to a significance level of 5%. SPSS 17 (SPSS Inc., Chicago, IL, USA) software was used for all of the statistical calculations.

## 3. Results

Table 1 presents the main characteristics of the study participants by groups of quality of life score. There were no significant differences in background characteristics by groups of quality of life score, with the exception of age, as, among participants scoring lower points in the quality of life score, there were a higher proportion of younger individuals (Table 1). Regarding the distribution of comorbidities, only prevalence of hypertension was significantly distributed differently across groups of quality of life score, with a slightly lower occurrence among individuals in the second quartile of quality of life (Table 1).

Table 2 shows the frequency distribution and association between quartiles of Mediterranean diet adherence score and the overall and individual domains of quality of life. A significant linear trend of association was found for overall quality of life (for the highest vs. the lowest quartile, OR = 10.01, 95% CI: 6.53, 15.33) and most of the domains besides satisfaction for “mental health” and “people with whom individual lives”, for which the association was stronger in the third quartile of the Mediterranean diet adherence score.

Table 3 shows the association between individual components of the Mediterranean diet adherence score and the overall and individual domains of quality of life score. All of the components were associated with overall quality of life, despite only fruit, vegetable, and legume being associated with nearly all domains of quality of life, while other components only partially.

## 4. Discussion

In this study, we reported a direct association between the adherence to the Mediterranean diet and quality of life measured with a multi-domain instrument. However, another component of the MANSA score result was significantly associated with a higher adherence to the Mediterranean diet. The results are generally in line with previously published studies [18,20,21,22,38,39], which reinforces the assumption that diet and overall wellbeing (measured as quality of life) are strictly related. When separately considering physical and mental health, we found an association for the physical component of the MANSA score, while no significant results were reported for mental health. These results in line with a study that was conducted in two cohorts of older adults, for which adherence to this dietary pattern was not associated with clinically relevant mental component summaries of the quality of life score used [21]. In contrast, other studies reported an association with mental, rather than physical, health [18]. Despite being unable to perform a direct quantitative comparison between existing evidence due to the differences in study design and methodology that are used to calculate either the adherence to the Mediterranean diet and the quality of life, it is important to identify at least whether a relation does exist, the potential determinants, and the possible mechanisms that underlie it.

High adherence to a Mediterranean dietary pattern has been associated with a longer lifespan and decreased risk of numerous non-communicable diseases, including cardio-metabolic disorders and certain cancers [13]. Previous findings from this cohort showed that individuals that are highly adherent to this dietary pattern were less likely to suffer from obesity [40], hypertension [41], and dyslipidemias [42]; however, in this study, we found that, also after adjustment for clinical status, a high adherence to the Mediterranean diet was associated with a better quality of life when compared to low adherence. Regarding elderly individuals, the Mediterranean diet has also been reported to exert benefits toward depression [11], neurocognitive disorders, and cognitive decline [12], leading to an overall better mental health. In line with this, a number of studies have reported that, not only Mediterranean diet being understood as dietary pattern, but also individual components of the Mediterranean diet, might be associated with better physical and mental health. Specifically, there is a large number of studies reporting a decreased risk of non-communicable diseases associated with higher intake of fish, olive oil [43], fruit and vegetable [44], nuts and seeds [45], as well as with limited consumption of animal proteins and excessive alcohol consumption [46,47]. In addition, some evidence explored the association between fruit and vegetable [48], fish [49], and nuts [50], and specifically quality of life in the general population, despite that results being contrasting and the overall body of literature being focused on this outcome remaining scarce. Moreover, individual clinical trials that specifically focus on one aspect (i.e., low fat diets) did not lead to significant results, suggesting that the prescription of restrictive diets in patients may be interpreted as a different experience with potential detrimental effects [51]. Thus, it is auspicial that dietary interventions on qualitative outcomes, such as quality of life, would benefit from healthy and palatable alternatives, especially those that are a part of the traditional dietary patterns and cultural heritage of a population.

From a mechanistic point of view, interactions between various foods and nutrients occur in the real-world nutrition and the overall variety of a diet might affect the health of individuals through a synergistic action of all its components. Antioxidant micro-nutrients and phytochemicals, such as polyphenols, which are highly contained in the Mediterranean diet, have been hypothesized to exert, at least in part, the potential beneficial effects on physical and mental health of individuals, leading to an overall better quality of life. There is evidence that the Mediterranean diet may also protect from depressive disorders and improve mental health through mechanisms that are related to inflammation, besides the aforementioned association with cardio-metabolic diseases and cancers. The most studied compounds that potentially mediate such effects are omega-3 polyunsaturated fatty acids (mainly derived from fish), monounsaturated fatty acids (mainly derived from olive oil), antioxidant vitamins including, but are not limited to, B-vitamins, vitamin D, vitamin A, and vitamin E (mainly derived from fruit and vegetable) and fiber (also derived from whole-grains), which have been reported to reduce the low-grade inflammatory status and improve the endothelial function and the profile of coagulation and inflammation biomarkers. Finally, results of recent research showed that a number of phytochemical compounds, such as polyphenols, have been associated with various health outcomes, such as a decreased risk of cardiovascular diseases [52], diabetes, hypertension, and metabolic disorders [53,54,55], depression [56], certain cancers [57], and overall prolonged lifespan [58]. Some evidence of the potentially beneficial effects of some polyphenol classes, such as flavonoids, phenolic acids, and phytoestrogens, have been reported in the same population that was included in this study [8,59]. However, further research is needed to better understand whether these phytochemicals may play a role in the overall health and quality of life of individuals.

Besides the biological connection between the dietary patterns and health, a demonstration of the association between adherence to the Mediterranean diet and quality of life may be part of better cultural background or economic allowance that may lead to a cluster of factors that characterize a healthier lifestyle [60]. On one side, there is evidence that higher adherence to the Mediterranean diet is associated with higher socio-economic status and better income, even in Mediterranean countries [61,62,63]. On the other, this association is not consistent over the whole Italian territory and other evidence showed that the adherence to this dietary pattern is rather associated with higher cultural status, suggesting that, in certain areas (including the same population on which was conducted the present study), the adherence to the Mediterranean diet is a matter that is related to the cultural heritage rather than economical allowance [64,65,66]. Besides, in this study, we did not find any significant association between educational and occupational status and quality of life, suggesting that such variables are rather not confounding the association with the dietary factors investigated. Thus, we hypothesize that other aspects that are related to quality of life may be affected by strong bond with cultural heritage characteristic of the Mediterranean area: psychosocial aspects of lifestyle should be addressed, such as family and community support, engagement in social activities, and conviviality are directly linked with health [67]. Some studies have shown evidence regarding the association of socialization (in terms of social networks and social engagement) and cardiovascular [68] and cognitive health [69]. Those individuals that are more adherent to the Mediterranean diet may have also social interactions, such as mealtime conversations, family leisure activities, or other forms of social engagement, which in turn resulted in better quality of life. 

The results of this study should be considered in light of some limitations. The most important issue that is common to all studies investigating quality of life, as outcome is the potential reverse causation. Indeed, the cross-sectional nature of the study does not allow for identifying a causal relationship. Among other limitations, even though a trained healthcare worker collects dietary information (either medical doctor, nurse, or nutritionist), data from a FFQ may be subjected to recall bias.

## 5. Conclusions

In conclusion, higher adherence to the Mediterranean diet was associated with a higher quality of life in adults living in Southern Italy. The results from this study support further investigation that examines the relation between dietary factors and quality of life with better methodological design (i.e., prospective studies). Further evidence is needed to better understand the relation between such life aspects and to plan educational programs that are to improve both dietary aspects and the quality of life of the general population.

## Figures and Tables

**Table 1:** Baseline characteristics of the study participants by groups * of quality of life score.

	MANSA Score				
	Group 1	Group 2	Group 3	Group 4	*p*-value
**Sex**					0.814
Men	200 (42.4)	196 (39.9)	222 (41.3)	186 (42.8)	
Women	272 (57.6)	295 (60.1)	316 (58.7)	249 (57.2)	
**Age groups *n*, (%)**					0.002
<39	172 (36.4)	206 (42.0)	169 (31.4)	136 (31.3)	
40–59	167 (35.4)	161 (32.8)	184 (34.2)	169 (38.9)	
>60	133 (28.2)	124 (25.3)	185 (34.4)	130 (29.9)	
**Educational status**					0.578
Low	179 (37.9)	162 (33.0)	203 (37.7)	153 (35.2)	
Medium	166 (35.2)	186 (37.9)	201 (37.4)	167 (38.4)	
High	127 (26.9)	143 (29.1)	134 (24.9)	115 (26.4)	
**Occupational status**					0.442
Unemployed	111 (26.2)	121 (30.0)	132 (29.5)	97 (25.3)	
Low	73 (17.2)	55 (13.6)	75 (16.8)	63 (16.4)	
Medium	126 (29.7)	103 (25.5)	111 (24.8)	100 (26.1)	
High	114 (26.9)	125 (30.9)	129 (28.9)	123 (32.1)	
**Smoking status**					0.775
Never smoker	284 (60.2)	314 (64.0)	337 (62.6)	260 (59.8)	
Former smoker	115 (24.4)	116 (23.6)	124 (23.0)	110 (25.3)	
Current smoker	73 (15.5)	61 (12.4)	77 (14.3)	65 (14.9)	
**Physical activity level**					0.104
Low	82 (19.4)	87 (19.8)	89 (18.6)	71 (18.3)	
Moderate	227 (53.7)	226 (51.4)	222 (46.4)	181 (46.8)	
High	114 (27.0)	127 (28.9)	167 (34.9)	135 (34.9)	
**Comorbidities**					
Hypertension, *n* (%)	238 (50.4)	223 (45.4)	278 (51.7)	237 (54.5)	0.043
Diabetes, *n* (%)	30 (6.4)	34 (6.9)	43 (8.0)	39 (9.0)	0.450
Dyslipidemia, *n* (%)	87 (18.4)	87 (17.7)	98 (18.2)	84 (19.3)	0.939
Cardiovascular diseases, *n* (%)	38 (8.2)	37 (7.8)	41 (7.9)	38 (8.9)	0.921
Cancer, *n* (%)	21 (4.4)	11 (2.2)	30 (5.6)	16 (3.7)	0.052
Obesity, *n* (%)	68 (16.2)	82 (18.0)	101 (20.0)	66 (15.9)	0.332

* Groups were defined as quartiles of MANSA.

**Table 2:** Distribution and association between overall quality of life and individual domain satisfaction of the study participants and Mediterranean diet adherence score (participants were grouped into quartiles of the score).

	Mediterranean Diet Score, OR (95% CI)				
	Q1	Q2	Q3	Q4	*P for trend*
Overall QoL *	52 (11.3)	36 (6.1)	190 (31.4)	157 (57.1)	
OR (95% CI) ^#^	1	0.42 (0.24, 0.71)	3.66 (2.52, 5.30)	10.01 (6.53, 15.33)	<0.001
Life as a whole, *n* (%) ^§^	94 (20.3)	172 (29.0)	333 (55.0)	143 (52.0)	
OR (95% CI) ^#^	1	1.53 (1.09, 2.13)	5.04 (3.67, 6.93)	4.14 (2.83, 6.06)	0.142
Job (when having one), *n* (%) ^§^	71 (15.4)	140 (23.6)	234 (38.6)	120 (43.6)	
OR (95% CI) ^#^	1	1.52 (1.06, 2.19)	3.40 (2.42, 4.76)	4.37 (2.94, 6.49)	<0.001
Financial situation, *n* (%) ^§^	71 (15.4)	102 (17.2)	140 (23.1)	91 (33.1)	
OR (95% CI) ^#^	1	1.13 (0.77, 1.64)	1.65 (1.16, 2.35)	2.83 (1.89, 4.25)	<0.001
Amount and quality of friends, *n* (%) ^§^	143 (31.0)	224 (37.8)	329 (54.3)	149 (54.2)	
OR (95% CI) ^#^	1	1.23 (0.91, 1.66)	2.39 (1.79, 3.19)	2.40 (1.68, 3.43)	0.056
Leisure activities, *n* (%) ^§^	96 (20.8)	131 (22.1)	229 (37.8)	98 (35.6)	
OR (95% CI) ^#^	1	0.91 (0.64, 1.29)	2.00 (1.46, 2.75)	1.74 (1.18, 2.57)	<0.001
Housing, *n* (%) ^§^	168 (36.4)	291 (49.1)	336 (55.4)	154 (56.0)	
OR (95% CI) ^#^	1	1.94 (1.45, 2.59)	2.27 (1.70, 3.02)	2.34 (1.64, 3.34)	<0.001
Personal safety, *n* (%) ^§^	170 (36.8)	190 (32.0)	296 (48.8)	163 (59.3)	
OR (95% CI) ^#^	1	0.74 (0.55, 1.00)	1.65 (1.24, 2.19)	2.37 (1.66, 3.37)	<0.001
People with whom individual lives, *n* (%) ^§^	21 (45.9)	400 (67.5)	494 (81.5)	173 (62.9)	
OR (95% CI) ^#^	1	2.64 (1.96, 3.54)	5.59 (4.05, 7.72)	1.98 (1.39, 2.84)	0.293
Sex life, *n* (%) ^§^	124 (26.8)	231 (39.0)	325 (53.6)	132 (48.0)	
OR (95% CI) ^#^	1	1.89 (1.38, 2.57)	3.51 (2.59, 4.75)	2.79 (1.93, 4.03)	0.254
Relationship with family, *n* (%) ^§^	237 (51.3)	434 (73.2)	467 (77.1)	171 (62.2)	
OR (95% CI) ^#^	1	2.87 (2.11, 3.89)	3.14 (2.32, 4.26)	1.62 (1.13, 2.32)	0.178
Physical health, *n* (%) ^§^	146 (31.6)	191 (32.2)	335 (55.3)	138 (50.2)	
OR (95% CI) ^#^	1	0.86 (0.63, 1.17)	2.62 (1.96, 3.51)	2.13 (1.49, 3.05)	0.652
Mental health, *n* (%) ^§^	233 (50.4)	382 (64.4)	514 (84.8)	161 (58.5)	
OR (95% CI) ^#^	1	1.87 (1.40, 2.50)	5.31 (3.82, 7.37)	1.36 (0.95, 1.93)	0.542

* an overall satisfaction with overall quality of life was arbitrarily defined as being in the highest quartile of the MANSA score (>70 points); **^§^** answers reflecting satisfaction of the domains were “pleased” and “couldn’t be better”; **^#^** adjusted for age (continuous), sex (male/female), BMI (<25 kg/m^2^, 25-30 kg/m^2^, >30 kg/m^2^), physical activity (low/medium/high), educational status (low/medium/high), occupational status (unemployed/low/medium/high), smoking status (current/former/never), alcohol consumption (no/moderate/regular), health status (presence of hypertension, type-2 diabetes, dyslipidaemias, cardiovascular disease, cancer), and total energy intake.

**Table 3:** Association between overall quality of life and individual domain satisfaction **^§^** of the study participants and individual components of the Mediterranean diet score.

	Mediterranean Diet Score Components, OR (95% CI)								
	Fruit	Vegetable	Legume	Dairy	Whole-grain	Fish	Meat	Olive oil	Alcohol
Overall QoL *	1.99 (1.54, 2.57)	2.14 (1.66, 2.75)	2.53 (1.92, 3.33)	1.90 (1.47, 2.47)	1.66 (1.29, 2.13)	1.80 (1.38, 2.35)	1.50 (1.13, 1.97)	1.53 (1.19, 1.98)	1.82 (1.23, 2.69)
Life as a whole	1.81 (1.46, 2.26)	1.52 (1.22, 1.89)	2.01 (1.60, 2.52)	1.40 (1.13, 1.75)	1.39 (1.11, 1.73)	1.45 (1.16, 1.80)	1.13 (0.89, 1.43)	1.28 (1.03, 1.59)	1.44 (1.00, 2.09)
Job (when having one)	1.61 (1.28, 2.03)	1.46 (1.16, 1.84)	1.53 (1.21, 1.95)	1.33 (1.05, 1.68)	1.29 (1.02, 1.63)	1.43 (1.13, 1.81)	1.31 (1.02, 1.69)	1.61 (1.28, 2.04)	2.05 (1.41, 2.97)
Financial situation	1.67 (1.29, 2.16)	1.21 (0.94, 1.56)	1.58 (1.21, 2.06)	1.19 (0.92, 1.54)	1.04 (0.80, 1.35)	1.11 (0.85, 1.43)	1.18 (0.89, 1.55)	1.22 (0.94, 1.57)	1.46 (0.97, 2.20)
Amount and quality of friends	1.19 (0.96, 1.46)	1.47 (1.19, 1.82)	1.38 (1.12, 1.72)	1.19 (0.97, 1.48)	1.13 (0.91, 1.41)	1.44 (1.16, 1.79)	1.19 (0.95, 1.50)	1.12 (0.91, 1.38)	1.60 (1.11, 2.31)
Leisure activities	1.30 (1.02, 1.64)	1.44 (1.14, 1.82)	1.22 (0.96, 1.55)	1.33 (1.05, 1.69)	1.03 (0.81, 1.31)	1.10 (0.86, 1.39)	1.06 (0.83, 1.37)	1.04 (0.82, 1.31)	1.54 (1.05, 2.27)
Housing	1.25 (1.02, 1.54)	1.21 (0.98, 1.49)	1.57 (1.27, 1.94)	1.20 (0.97, 1.48)	1.12 (0.90, 1.38)	1.50 (1.21, 1.85)	0.96 (0.77, 1.20)	1.16 (0.94, 1.43)	1.41 (0.97, 2.04)
Personal safety	1.26 (1.02, 1.56)	1.17 (0.94, 1.44)	1.54 (1.24, 1.92)	1.30 (1.05, 1.61)	1.18 (0.95, 1.46)	1.27 (1.03, 1.58)	1.30 (1.03, 1.63)	0.99 (0.80, 1.22)	1.16 (0.81, 1.68)
People with whom individual lives	1.70 (1.36, 2.13)	1.37 (1.10, 1.72)	1.98 (1.58, 2.48)	1.18 (0.95, 1.48)	1.05 (0.84, 1.32)	1.38 (1.11, 1.73)	1.01 (0.80, 1.28)	1.09 (0.87, 1.36)	2.34 (1.47, 3.71)
Sex life	1.46 (1.18, 1.81)	1.43 (1.16, 1.77)	1.74 (1.40, 2.17)	1.11 (0.89, 1.37)	1.25 (1.01, 1.56)	1.51 (1.21, 1.88)	1.09 (0.86, 1.37)	1.26 (1.02, 1.56)	1.24 (0.86, 1.79)
Relationship with family	1.36 (1.09, 1.70)	1.34 (1.07, 1.67)	1.60 (1.28, 2.00)	1.09 (0.87, 1.36)	1.06 (0.85, 1.34)	1.16 (0.93, 1.46)	1.09 (0.86, 1.38)	1.29 (1.03, 1.62)	1.66 (1.08, 2.55)
Physical health	1.47 (1.18, 1.81)	1.35 (1.09, 1.67)	1.44 (1.16, 1.79)	1.34 (1.08, 1.66)	1.22 (0.98, 1.52)	1.16 (0.93, 1.44)	1.27 (,1.01, 1.59)	1.18 (0.95, 1.46)	1.09, 0.75, 1.58)
Mental health	1.34 (1.08, 1.68)	1.44 (1.15, 1.79)	1.46 (1.17, 1.82)	1.01 (0.81, 1.26)	0.99 (0.79, 1.24)	1.17 (0.94, 1.47)	1.16 (0.91, 1.46)	1.45 (1.16, 1.81)	1.24 (0.83, 1.85)

^§^ answers reflecting satisfaction of the domains were “pleased” and “couldn’t be better”. * an overall satisfaction with overall quality of life was arbitrarily defined as being in the highest quartile of the MANSA score (>70 points).
